# Ammonia metabolic reprogramming in the tumor microenvironment: emergence of an immunosuppressive niche

**DOI:** 10.3389/fcell.2026.1826809

**Published:** 2026-06-22

**Authors:** Zihao Ye, Hao Wu, Zhanhao Li, Ruizhe Ye, Yingliang Rao, Bin Liu, Baoshan Gao

**Affiliations:** 1 Department of Urology II, The First Hospital of Jilin University, Changchun, China; 2 School of Basic Medicine, Tongji Medical College, Huazhong University of Science and Technology, Wuhan, China; 3 Department of Urology, China-Japan Hospital of Jilin University, Changchun, China

**Keywords:** ammonia metabolism, cancer, metabolic reprogram, SLC (solute carrier family), TME (tumor microenvironment)

## Abstract

Metabolic reprogramming within the tumor microenvironment plays a pivotal role in tumor proliferation, progression, and immune evasion. Cancer cells exhibit altered lipid, glucose, and amino acid metabolism to adapt to hostile conditions such as hypoxia and nutrient deprivation. Particularly, ammonia metabolism has emerged as a critical aspect of tumor metabolic reprogramming. Oncogene mutations, such as those in *c-MYC*, *KRAS*, and *p53*, regulate key enzymes involved in amino acid metabolism, which in turn affects tumor cell survival and proliferation. Elevated ammonia levels in the TME (Tumor Microenvironment) not only provide essential nitrogen for cell growth but also impair immune cell function, including T cells and natural killer cells, contributing to immune evasion. High ammonia concentrations suppress T cell activation and promote exhaustion, while interfering with natural killer cell cytotoxicity by hindering perforin maturation. Moreover, ammonia accumulation fosters an immunosuppressive microenvironment, influencing cytokine secretion and facilitating tumor metastasis. Targeting ammonia metabolism, in combination with immune checkpoint inhibitors, presents a promising therapeutic strategy to enhance immune responses and inhibit tumor progression. This review consolidates recent findings on the role of ammonia metabolism in the TME, highlighting its potential as a therapeutic target to improve cancer treatment outcomes.

## 1 Introduction

Metabolic reprogramming in the tumor microenvironment (TME) refers to the alterations in various energy metabolism pathways in cancer cells, primarily involving changes in lipid, glucose, and amino acid metabolism (Martínez-Re et al., 2021). Recently, our understanding of metabolic reprogramming has evolved from focusing solely on individual cancer cells to exploring the relationship between tumor metastasis, the tumor microenvironment, and metabolic alterations. Notably, by-products of metabolic reprogramming in cancer cells have found widespread clinical applications, such as hyperpolarized 13C-pyruvate Magnetic Resonance Imaging (MRI) and targeted metabolic therapies like glutamine inhibitors (Woitek and Brindle, 2023; Yang et al., 2021).

**FIGURE 1:**
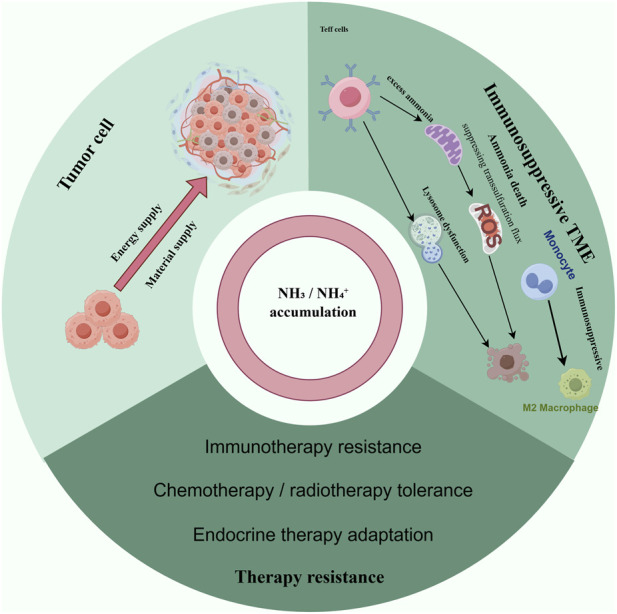
Interaction Between tumor cell ammonia metabolism and TME. (Tumor ammonia metabolic reprogramming promotes accumulation of ammonia (NH_3_/NH_4_^+^) in the tumor microenvironment. Concurrently, ammonia imposes profound effects on infiltrating immune cells, reshaping the TME toward an immunosuppressive state. In effector T cells, excess ammonia disrupts lysosomal and mitochondrial function, elevates reactive oxygen species (ROS), and compromises bioenergetic fitness, resulting in impaired activation, functional exhaustion, and cell death. In parallel, ammonia-driven metabolic rewiring of myeloid cells promotes polarization toward immunosuppressive M2-like macrophages, further dampening antitumor immune responses. Together, ammonia-induced immune dysfunction and myeloid reprogramming establish a permissive immunosuppressive niche that constrains effective immune surveillance. Together, these effects facilitate immune evasion and reduce responsiveness to immunotherapy. In parallel, ammonia-associated rewiring supports tumor survival under stress, contributing to resistance to chemotherapy, radiotherapy, and endocrine therapy. Figure created by Figdraw).

Previous studies primarily concentrated on glucose metabolism, particularly aerobic and anaerobic respiration. However, glucose metabolism in cancer cells exhibits limited heterogeneity, providing relatively few therapeutic targets (Hindes et al., 2025; Liberti and Locasale, 2016). In recent years, research has shifted toward amino acid metabolism, a crucial process involved in various cellular metabolic pathways. Under nutrient-deprived conditions, intermediates of amino acid metabolism, such as glutamine and glutamate, play essential roles in cancer cell growth and proliferation (Li et al., 2023). Unlike glucose and lipid metabolism, glutamine metabolism not only supplies energy but also provides precursors for the synthesis of macromolecules required for cell survival, acting as a bridge between energy metabolism and cell proliferation (Altman et al., 2016; Tardito et al., 2015). Moreover, there has been limited research on how amino acid metabolic reprogramming interacts with the diverse components of the tumor microenvironment, particularly immune cells that infiltrate the tumor. This review draws upon the literature from the past decade to systematically analyze how ammonia metabolic reprogramming shapes an immune-suppressive, metabolism-supportive tumor microenvironment landscape, thereby promoting tumor initiation and progression. Additionally, we discuss potential therapeutic targets that could aid in advanced cancer therapy. More detailed information can be found in Figure 1.

## 2 Ammonia metabolism in normal cells

Ammonia metabolism in the body involves three key processes: generation, transport, and excretion, which are essential for maintaining nitrogen balance, bio-synthesis and preventing ammonia toxicity (Huang et al., 2021). Ammonia is primarily generated through the deamination of amino acid, particularly in the glutamate-glutamine cycle, where glutamate dehydrogenase catalyzes the removal of an amino group from glutamate to produce free ammonia. Additionally, ammonia arises from the catabolism of branched-chain amino acid and from gut bacterial breakdown of dietary proteins. Due to its toxicity, ammonia is transported in less toxic forms, such as glutamine and alanine, which are synthesized in tissues and transported to the liver and being used in urea cycle to excrete as urea (Feng et al., 2018). In the liver, ammonia is converted into urea through the urea cycle, which involves several enzymes, including carbamoyl phosphate synthetase I (CPS I), ornithine transcarbamylase (OTC), and arginase. Intracellular ammonia metabolism is predominantly associated with the metabolic processes of various amino acid. As a downstream pathway of amino acid metabolism, ammonia metabolism facilitates the conversion of nitrogen elements derived from biological macromolecules into urea. Urea is then transported to the kidneys for excretion (Ilaiwy et al., 2019). The urea cycle not only facilitates ammonia clearance but also supports critical metabolic processes, including nucleic acid synthesis, gluconeogenesis, and energy production, ensuring overall physiological homeostasis. Gut microbiota also contributes to systemic ammonia homeostasis by regulating intestinal ammonia production, barrier integrity, and microbial metabolite profiles (Morais et al., 2021). Dysbiosis, particularly enrichment of urease-producing bacteria, can increase ammonia generation and facilitate its entry into the circulation (Wang et al., 2023). Conversely, a balanced microbiota and short-chain fatty acid production may help maintain intestinal barrier function, reduce ammonia absorption, and indirectly support hepatic ammonia detoxification (Wang et al., 2019). Thus, microbiota-derived ammonia may represent an additional source of systemic nitrogen burden, although its direct contribution to ammonia accumulation within the tumor microenvironment remains to be further defined.

## 3 Role of ammonia metabolism in tumor cells

### 3.1 Ammonia metabolic reprogram provides energy and material for tumor proliferation

**FIGURE 2:**
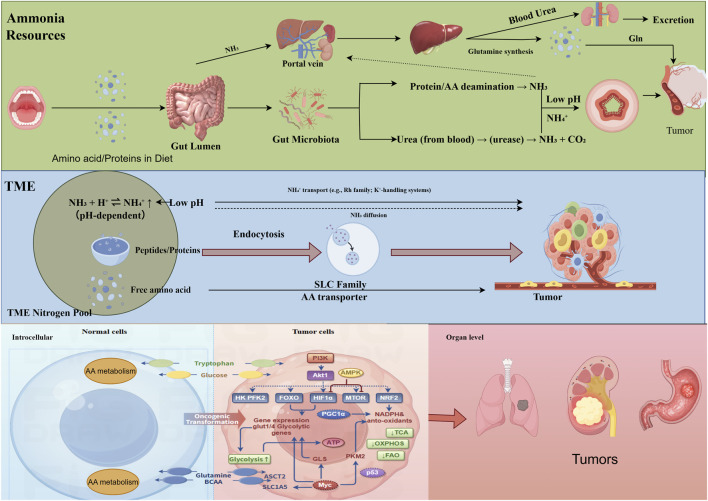
Ammonia metabolic reprogramming in tumor cells, TME and organ level. (At the cellular level, ammonia is predominantly generated through amino acid metabolism, particularly glutaminolysis and amino acid deamination accompanying TCA cycle anaplerosis. Glutamine is converted to glutamate by glutaminase, followed by entry into the TCA cycle *via* glutamate dehydrogenase or transamination reactions, with concomitant release of free ammonia (NH_3_/NH_4_^+^). In many tumor cells, reduced expression or activity of key urea cycle enzymes constrains ammonia detoxification, leading to sustained intracellular ammonia accumulation. Rather than being eliminated, ammonia is actively reassimilated through glutamine synthetase–mediated nitrogen fixation, coupling nitrogen availability to nucleotide biosynthesis, nonessential amino acid production, and redox-supportive anabolic programs. This intracellular ammonia recycling is tightly integrated with rewired carbon metabolism, including enhanced glycolytic flux, altered TCA cycle activity, mitochondrial oxidative phosphorylation, and fatty acid oxidation, thereby supporting bioenergetic flexibility and redox homeostasis under nutrient stress. At the tumor microenvironment level, continuous metabolic activity and high cellular turnover establish a nitrogen-rich niche characterized by elevated ammonia availability. Acidic pH drives pH-dependent interconversion between NH_3_ and NH_4_^+^, enabling passive NH_3_ diffusion across membranes and transporter-mediated NH_4_^+^ flux *via* Rh family or potassium-associated transport systems. In parallel, tumor cells acquire extracellular nitrogen through macropinocytosis of proteins and uptake of free amino acid *via* SLC-family transporters, further amplifying local nitrogen recycling. Intracellular ammonia metabolism functions as a central metabolic node that links oncogenic signaling to metabolic reprogramming. PI3K–Akt–mTOR signaling promotes glutamine utilization, amino acid uptake, and anabolic flux, whereas AMPK integrates energetic stress with nitrogen recycling and mitochondrial metabolism. Hypoxia-driven HIF-1α signaling reshapes ammonia-linked carbon flux by coordinating glycolysis and mitochondrial function, while NRF2 couple ammonia metabolism to redox control through regulation of antioxidant and amino acid biosynthetic pathways. In parallel, p53 signaling modulates nitrogen handling by constraining anabolic demand and preserving mitochondrial integrity. Together, signaling-controlled ammonia recycling synchronizes nitrogen flux with glycolysis, TCA cycle activity, oxidative phosphorylation, and fatty acid oxidation, establishing a permissive metabolic state that supports tumor growth, metabolic plasticity, immune evasion, and therapeutic resistance).

The metabolism of tumor cells differs substantially from that of normal cells. Tumor cells generally proliferate rapidly. In contrast, the oxygen levels in the tumor microenvironment are insufficient to meet the demands of both cell proliferation and metabolism. For glucose metabolism, the Warburg effect represents a mechanism through which tumor cells acquire nutrients and generate energy by converting glucose into pyruvate, which is then further converted into lactate *via* anaerobic glycolysis to produce ATP (Zhong et al., 2022; Warburg, 1956). When energy from aerobic respiration being inhibited, glutamine metabolism is a critical energy source for tumor cells. The α-ketoglutarate (α-KG) produced by deamination of glutamate or other amino acid during the final stage of ammonia metabolism can directly feed into the TCA (Tricarboxylic Acid) cycle of tumor cells (Alam et al., 2016). Glutamine catabolism generates glutamate, α-ketoglutarate, and ammonia, thereby linking carbon metabolism with nitrogen recycling in cancer cells. Under glutamine-depleted conditions, selected cancer cells can reassimilate ammonia through GDH/GDH2-mediated reductive amination to produce glutamate and downstream nitrogen-containing metabolites, including proline and aspartate (Cluntun et al., 2017; Márquez et al., 2006). ^15^N-tracing studies further support this ammonia-to-glutamate flux and show that ammonia assimilation is markedly impaired in GDH-deficient cells (Matés et al., 2020). However, ammonia utilization is highly cell-type-specific: MCF7 and T47D breast cancer cells can use ammonia to support proliferation, whereas osteosarcoma and HepG2 cells show limited proliferative responses (Spinelli et al., 2017; Roberts et al., 2012; Sakr et al., 2018). Therefore, ammonia should be regarded as a context-dependent alternative nitrogen source rather than a universal requirement for cancer-cell survival and proliferation (Tardito et al., 2015). More information can be found in Figure 2.

Although ammonia can serve as an alternative nitrogen source under glutamine-depleted conditions, this capacity is highly cell-type-specific and remains mechanistically incompletely defined. Selected cancer models, including HEK293, Huh7, T47D, and MCF7 cells, can utilize ammonia to support proliferation, whereas other cells such as LNCAP and MCF10A may exhibit limited utilization or ammonia-induced toxicity (Lie et al., 2019; Takeuchi et al., 2018). This heterogeneity may be determined by several interconnected factors, including GDH isoform activity, SLC4A11-mediated ammonia transport, GLUL-dependent glutamine synthesis, and downstream AMPK–mTORC1 signaling (Dasarathy et al., 2017). Moreover, ammonia exposure may lead to divergent biological outcomes across tumor types; for example, it can support proliferation in some breast cancer cells, whereas in hepatocellular carcinoma models it may transiently suppress early growth but subsequently enhance stemness and metastatic potential after ammonia withdrawal (Tardito et al., 2015; Adeva et al., 2012; DeBerardinis et al., 2007; Harder et al., 2014). Therefore, ammonia utilization should be interpreted as a context-dependent metabolic adaptation rather than a universal feature of cancer metabolism. Further studies integrating isotope-tracing, transporter perturbation, and patient-derived tumor models are needed to define which tumors are truly ammonia-dependent.

Ammonia-related nitrogen metabolism may also intersect with redox adaptation by influencing glutamate availability, a key substrate for glutathione synthesis. In tumor cells, glutamate-cysteine ligase and glutathione synthetase catalyze GSH biosynthesis from glutamate, cysteine, and glycine (Franklin et al., 2009). In KEAP1-deficient or NRF2-activated tumors, transcriptional upregulation of GSH-related genes such as GCLC, GCLM, and GSR, together with increased NADPH availability, enhances antioxidant capacity (Lien et al., 2016). Oncogenic induction of the cystine/glutamate antiporter SLC7A11 further supports cysteine uptake and GSH production (Jiang et al., 2015). This redox program may help tumor cells tolerate hypoxia-induced ROS accumulation and oxidative stress (Chandel et al., 1998). However, the contribution of ammonia itself to GSH-dependent redox homeostasis should be distinguished from the broader effects of glutamine metabolism, glutamate availability, and cystine transport.

### 3.2 Ammonia metabolism activates SREBPs (sterol regulatory element-binding protein) synthesis and lipid metabolism in tumor cell

**FIGURE 3:**
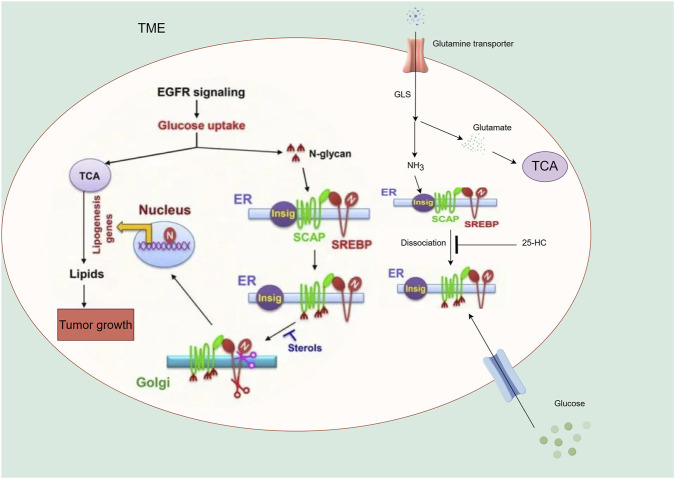
Ammonia regulates the expression of lipid-related genes by promoting the formation of the SCAP (SREBP cleavage-activating protein)/SREBP complex. (Tumor cells coordinate growth factor signaling and nutrient metabolism to couple carbon and nitrogen flux with lipid biosynthesis. EGFR signaling enhances glucose uptake, thereby fueling glycolysis and TCA cycle activity and supporting N-glycan synthesis required for proper maturation of membrane-associated proteins. In parallel, glutamine uptake and glutaminase-mediated glutaminolysis generate glutamate and free ammonia (NH_3_/NH_4_^+^), contributing to TCA cycle anaplerosis and intracellular nitrogen flux. Accumulation of ammonia reflects sustained amino acid catabolism and functions as a metabolic input that integrates nitrogen availability with biosynthetic demand. Metabolic signals converge at the endoplasmic reticulum (ER), where SREBPs are retained in an inactive state through interaction with SCAP and Insig (insulin-induced gene) under sterol-replete conditions. Changes in metabolic state and sterol composition promote dissociation of the SCAP–SREBP complex from Insig, enabling ER-to-Golgi trafficking and proteolytic activation of SREBPs. Activated SREBPs translocate to the nucleus to induce transcription of lipogenic and metabolic genes, thereby coupling glucose- and glutamine-derived metabolic flux, including ammonia production, to lipid synthesis. Feedback inhibition by sterols and oxysterols, such as 25-hydroxycholesterol, constrains excessive SREBP activation. Through coordinated regulation of glutamine-derived ammonia production, carbon metabolism, and ER–Golgi lipid sensing, tumor cells establish an anabolic metabolic program that supports tumor growth within the tumor microenvironment. Reprinted from Cancer cell, Vol 5, Chunming Chen.et.al, Glucose-Mediated N-glycosylation of SCAP Is Essential for SREBP-1 Activation and Tumor Growth, Pages No 1., Copyright (2015), with permission from Elsevier).

Recent studies have shown that glutamine promotes lipogenesis and activates sterol regulatory element-binding proteins including SREBP-1a, SREBP-1c and SREBP-2 (Zhong et al., 2024). The combination of glutamine and glucose activates the cleavage of SREBP-1 and SREBP-2, which enhances fatty acid and cholesterol synthesis, promoting cancer cell proliferation (Geng et al., 2023). Ammonia produced by glutaminase (GLS) during glutamine metabolism activates SREBPs, mainly SREBP-1, key transcription factor that regulate lipid synthesis. Lipogenesis is crucial for tumor progression as it supports both the proliferation and survival of cancer cells (Cheng et al., 2020; Kou et al., 2022). Studies show that ammonia, rather than other glutamine metabolites like glutamate or α-ketoglutarate, is responsible for activating SREBP cleavage and promoting lipogenesis (Geng and Guo, 2024). This mechanism involves ammonia binding to SCAP, inducing a conformational change that causes SCAP to dissociate from its inhibitory protein Insig (Cheng et al., 2022; Ya et al., 2002). This transcriptionally active fragment undergoes nuclear import and transactivates genes governing lipid metabolism. This dissociation step is essential for SREBP activation, which subsequently initiates lipogenesis (Espenshade et al., 2002). Studies also show that inhibiting GLS activity (through genetic methods or the GLS inhibitor CB-839) effectively blocks this process, preventing SREBP activation and lipogenesis (Adams et al., 2003). Research conducted by Chen et al. elucidates ammonia’s signaling role in tumor lipogenesis *via* SREBP activation (Cheng et al., 2022). The study reveals that ammonia released from glutamine triggers sequential conformational changes in N-glycosylated SCAP, driving its dissociation from Insig to enable SREBP translocation (Guo, 2016; Cheng et al., 2015; Lee et al., 2022). Crucially, an allosteric binding pocket within SCAP transmembrane domain defined by residues D428, S326, and S330, mediating ammonia sensing. This mechanism is antagonized by 25-hydroxycholesterol (25-HC), which sterically blocks ammonia access to its binding site, thereby stabilizing the SCAP-Insig complex and suppressing SREBP activation (Ya et al., 2002; Espenshade et al., 2002; Adams et al., 2003; Yabe et al., 2002). Functional data demonstrate that glutamine and glucose exhibit metabolic complementarities in inducing lipid metabolic genes, with glutamine-dependent SREBP transactivation observed across diverse cancer lineages. The proposed glutamine-ammonia-SCAP axis establishes ammonia as a lipid metabolic regulator beyond its conventional role as a nitrogen donor. This paradigm-shifting discovery simultaneously redefines ammonia’s signaling function and unveils novel aspects of 25-HC mediated SCAP regulation, as detailed in Figure 3. *In vitro*, researchers identified glutamine as a critical determinant of lipid metabolic remodeling. Transcriptomic profiling showed that provision of either glutamine or glucose alone was insufficient to induce genes involved in fatty-acid and cholesterol biosynthesis/uptake (e.g., *SREBF1/2*, *ACLY*, *ACACA*, *FASN*, *SCD1*, *HMGCR* and *LDLR*); robust activation occurred only when glutamine and glucose were provided together, whereas SCAP mRNA levels remained unchanged. Consistently, protein-level analyses across multiple cancer cell lines demonstrated that glucose alone triggers neither *SREBP-1/2* proteolytic processing and activation nor upregulated downstream lipogenic targets (*FASN*, *SCD1*). By contrast, in the presence of glucose, glutamine potently induced SREBP cleavage and nuclear translocation in a dose- and time-dependent manner, thereby engaging a lipogenic program, enhancing glucose-derived lipid synthesis, and promoting proliferation, with SREBP-1 being particularly essential for cell growth. Notably, cholesterol depletion, enforced EGFR–PI3K–Akt activation, or supplementation with other amino acid failed to activate SREBP under glutamine deprivation, underscoring a high degree of glutamine specificity. Mechanistically, although glucose-dependent SCAP N-glycosylation was required to maintain SCAP stability, this modification alone was insufficient to drive SREBP activation; in the absence of glutamine, SCAP remained stable, yet SREBP failed to accumulate in the nucleus and execute its transcriptional program. Collectively, these data position glutamine as an intrinsic metabolic hub that couple glucose metabolism to SREBP-dependent lipogenesis, providing a requisite metabolic context for rapid tumor-cell proliferation. Ammonia emerges as a signaling metabolite that orchestrates metabolic reprogramming in cancer cells. By coupling nitrogen utilization with lipid synthesis and redox control, ammonia integrates anabolic growth with stress tolerance, revealing a previously unappreciated regulatory layer of tumor metabolism and exposing a potential vulnerability to metabolic intervention (Cheng et al., 2022).

### 3.3 Key enzyme and solute carrier family manipulates ammonia metabolism in tumor

The solute carrier family of transport proteins involved in ammonia metabolism plays a crucial role in regulating the lifecycle of both cancer stem cells (CSCs) and tumor cells (Pochini et al., 2014). The SLC family generally serves as carriers for glutamine (Gln) transport into cells, with their numbers and density proportional to the intensity of ammonia metabolism (Pacifico et al., 2023). Studies have found that ammonia promotes CSC characteristics, such as liver spheroid formation and ALDH activity, *via* the expression of the transporter protein SLC4A11 (Elaimy et al., 2024). This further enhances liver CSC function, increasing tumor-initiating cell (TIC) proliferation and promoting the development and growth of hepatocellular carcinoma (HCC). Ammonia clearance (e.g., mainly by enhancing ornithine cycle) significantly reduces HCC growth and liver CSC characteristics. Therefore, ammonia clearance could serve as a novel therapeutic strategy. However, SLC can also limit the proliferation of tumor cells. Reduced expression of the Ornithine cycle transporter SLC25A15 in the SLC family correlates with poor clinical prognosis in HCC patients. HCC cells with low SLC25A15 expression exhibit reprogrammed energy metabolism, leading to elevated glutamine levels and conversion into fatty acids through reductive metabolism (Wang et al., 2024). Loss of SLC25A15 causes intracellular ammonia accumulation and excessive glutamine uptake, activating the mTORC1 signaling pathway and driving HCC growth and metastasis. Additionally, SLC25A15 regulates PD-L1 immune checkpoint expression (Byun et al., 2020). Tumors with low SLC25A15 expression are resistant to anti-PD-L1 therapy but show enhanced responses to immunotherapy when treated with BPTES (glutamine metabolism inhibitor). Studies further reveal that SLC25A15, by regulating glutamine metabolism, creates metabolic vulnerabilities in HCC cells, suggesting that blocking glutamine metabolism could offer a new therapeutic approach for HCC (Zhang Q. et al., 2024). Additionally, ammonia metabolism can affect tumor cell autophagy and apoptosis by regulating their cell cycle (Mavrogonatou et al., 2015). Studies have indicated that ammonia induces cellular senescence in HCC cells, with SLC4A11 facilitating ammonia excretion to counteract ammonia-induced senescence (Chee et al., 2021; Zhang et al., 2015). Specifically, ammonia upregulates senescence-associated markers in HCC cells, resulting in cell cycle arrest. β-Catenin activation enhances ammonia stability and excretion, inhibiting senescence and promoting cell proliferation. Further analysis demonstrates that β-catenin activation increases glutamine hydrolysis, enhancing ammonia production and promoting its utilization by upregulating GLUD1 (Glutamate Dehydrogenase) expression (Spinelli et al., 2017). Consequently, targeting SLC4A11 could be a potential strategy for treating β-catenin-mutant HCC.

Beyond glutamine-related transporters, selected SLC family members may also contribute to tumor nitrogen metabolism. In HCC, CPS1 deficiency has been associated with altered SLC1A3 expression and intracellular aspartate homeostasis, thereby influencing tumor proliferation and metastatic behavior. Mechanistically, CPS1 silencing may affect both lipid metabolism and SLC1A3-mediated aspartate transport, suggesting a context-dependent role of the CPS1–SLC1A3 axis in HCC progression (Chen et al., 2024). More broadly, SLC transporters can regulate the uptake and exchange of amino acids and other metabolites, thereby shaping tumor metabolic plasticity, redox balance, and signaling dependencies. However, these effects should not be generalized as direct regulation of ammonia metabolism by all SLC members. Instead, SLC-mediated metabolic remodeling should be interpreted according to transporter subtype, substrate specificity, tumor lineage, and metabolic context, as summarized in Table 1.

**TABLE 1:** The mechanisms by which SLCs regulate ammonia metabolism to promote tumor progression in different tumor cells.

SLC	Subtype	Cancer	Substrate	Mechanism	Study result	References
SLC14	SLC14A1	PCa	Urea	SLC14A1 overexpression inhibits cell migration by suppressing CDK1/CCNB1 and mTOR/MMP-9 pathways.	Low SLC14A1 expression correlates with high Gleason score and smaller tumor volume.	Ma et al. (2024)
		UTUC/UBUC		Nuclear SLC14A1 recruits HDAC1/SIN3A to suppress HK2, while low SLC14A1 enhances glycolysis for tumor cell proliferation.	Knockdown of SLC14A1 reduces tumor cell proliferation, migration, and viability (P < 0.05)	Chan et al. (2020)
SLC7	SLC7A1	osteosarcoma	Arg	SLC7A1 promotes tumor cell proliferation by enhancing arginine metabolism.	SLC7A1 silencing reduces tumor cell invasion, migration, and spheroid formation *in vitro* (P < 0.05)	Liao et al. (2025)
SLC1	SLC1A1	NSCLC	Gln/Asp	Upregulated SLC1A1 in NSCLC enhances GSH synthesis, boosting tumor cell antioxidative capacity.	shRNA-mediated SLC1A1 silencing reduces tumor cell number by 30% *in vitro* (P < 0.05)	Guo et al. (2021)
SLC13	SLC13A3	HCC	Asp/GSH	CTNNB1 mutation in HCC activates SLC7A5 *via* MYC regulation, increasing leucine levels.	SLC13A3 knockout mice show significantly reduced tumor volume and weight compared to controls (ANOVA, P < 0.0001).	Zhao et al. (2024a)
SLC1	SLC1A3	HCC	Asp	CPS1 sustains intracellular aspartate levels by enhancing METTL14-dependent m6A modification and stabilization of the aspartate transporter SLC1A3, thereby promoting extracellular aspartate uptake to support tumor cell proliferation while restraining invasion through inhibition of the PC-PLC/DAG/PKC signaling axis.	Wound-healing assays demonstrated that SLC1A3 silencing increased the proliferative capacity of HCC cells.	Chen et al. (2024)
SLC4	SLC4A11		NH_4_^+^/H^+^	SLC4A11 upregulation in HCC cancer stem cells sustains intracellular ammonia to reprogram nitrogen metabolism toward glutamine-dependent amino acid and nucleotide biosynthesis, thereby promoting stemness, tumor initiation, and progression.	ammonium chloride treatment increased hepatosphere number and diameter in HepG2 cells	Elaimy et al. (2024)
SLC25	SLC25A15		Ornithine	SLC25A15 downregulation disrupts urea cycle function and causes ammonia accumulation, which suppresses OGDHL expression and redirects glutamine metabolism toward reductive carboxylation and lipid synthesis, thereby enhancing energy production and promoting tumor progression.	In cell viability and colony formation assay, both overexpression and knockout of SLC25A15 inhibited the proliferation of HCC, whereas knockdown of SLC25A15 promoted HCC cell proliferation.	Zhang et al. (2024a)
SLC38	SLC38A7	GC	Gln/Asp	The METTL3/IGF2BPs axis promotes gastric cancer proliferation and metastasis by upregulating SLC38A7 *via* m6A RNA methylation.	Lentiviral silencing of SLC38A7 reduces cell migration (Transwell), viability, and ATP content in gastric cancer cells (P < 0.05)	Hua et al. (2024)
		PC	Gln/Asp	Endocytosis links mTORC1 to Gln and Asn, activating mTORC1 and promoting pancreatic cancer growth.	SLC38A7 mutant pancreatic cancer cells are smaller and exhibit reduced proliferation compared to wild-type cells (P < 0.001).	Meng et al. (2022)
	SLC38A5	glioma	Gln/Asp/His	MYC expression inhibits SLC38A5-mediated glutamine uptake, potentially reducing tumor cell growth.	MYC binding to the SLC38A5 promoter reduces SLC38A5 expression and glioma cell activity.	Wise et al. (2008)
	SLC38A2	Breast cancer	Gln/Ala/Gly	Inhibition of SLC38A2-mediated glutamine uptake may reduce tumor cell growth.	SLC38A2 silencing by RNA interference reduces tumor cell colony formation *in vitro*.	Jeon et al. (2015)
SLC5	SLC5A8	CRC	SCFA	SLC5A8 inhibits colon cancer cell aggregation, suppressing proliferation and metastasis.	SLC5A8 methylation silencing reduces colony formation in colon cancer cells (P < 0.05).	Li et al. (2003)
SLC6	SLC6A14	PC	Neutral AA	SLC6A14 efficiently facilitates amino acid uptake in tumor cells to meet synthesis demands.	a-MT inhibition of SLC6A14 reduces invasion, and shRNA silencing inhibits pancreatic cancer colony formation.	Coothankandaswamy et al. (2016)
SLC7	SLC7A11	Breast cancer	Gln/Cys	SLC7A11 promotes tumor cell proliferation and migration by regulating amino acid uptake to produce GSH, maintaining redox homeostasis.	*In vitro*, SLC7A11 inhibition reduces breast cancer cell viability and colony formation.	Yang and Yee (2014)
		glioma			SLC7A11 inhibition reduces glioma cell migration *in vitro*.	Tsuchihashi et al. (2016)
		GC			*In vivo*, intraperitoneal injection of SLC7A11 inhibitor reduces gastric cancer tumor weight.	Ishimoto et al. (2011)
SLC1	SLC1A5	PCa	L-Gln	SLC1A5 regulates L-glutamine uptake to sustain tumor cell metabolic needs.	*In vivo*, shRNA-mediated SLC1A5 silencing reduces tumor volume in mice by 15-fold (P < 0.001).	Wang et al. (2015)
		Hela Cell			SiRNA-mediated SLC1A5 silencing reduces tumor cell volume by 10%.	Nicklin et al. (2009)
SLC7 SLC3	SLC7A5 SLC3A2	Hela cell	L-Leu	SLC7A5/SLC3A2 may activate the mTOR pathway and promote tumor malignancy by co-transporting L-glutamine and L-leucine.	BCH inhibition of SLC7A5/SLC3A2 reduces mTOR activation and tumor cell volume.	

The table summarizes the regulation of amino acid uptake by different SLCs and provides a detailed overview of how various regulatory factors influence SLCs, thereby altering amino acid metabolism within tumor cells and subsequently mediating tumor progression.

GC, gastric cancer; PCa, Prostate cancer; PC, Pancreatic cancer; HCC, Hepatocellular carcinoma; NSCLC, Non-small cell lung cancer; UTUC, Upper tract urothelial carcinoma; UBUC, Urinary bladder urothelial carcinoma.

### 3.4 Oncogenic rewiring of ammonia metabolism

Ammonia metabolism may exert varying effects on cancer cells depending on genetic influences, with recent research highlighting the impact of *KRAS* mutations and *LKB1* loss on NSCLC cell metabolism, particularly the critical role of CPS1 in the cell (Kim et al., 2017). LKB1 inhibits CPS1 expression through AMPK, whereas CPS I expression is elevated in *KRAS/LKB1*-mutant NSCLC cells (Çeliktaş et al., 2017; Shackelford and Shaw, 2009). Studies have demonstrated that silencing CPS I results in pyrimidine depletion, purine accumulation, and DNA damage, thereby inhibiting cell proliferation (Nigro et al., 1989). Supplementing with exogenous pyrimidines reverses these effects and restores cell growth. *KRAS/LKB1*-mutant NSCLC cells depend on CPS I to maintain the pyrimidine pool, facilitating tumor growth. Silencing CPS I suppresses tumor growth *in vivo*, indicating that CPS I and associated metabolic pathways may present novel therapeutic targets for *KRAS/LKB1*-mutant lung cancer. Mechanistically, CPS1 is a mitochondrial enzyme that catalyzes the conversion of ammonia and bicarbonate into carbamoyl phosphate (CP), the rate-limiting step of the urea cycle. In normal cells, mitochondrial CP is primarily consumed by the urea cycle *via* ornithine transcarbamylase (OTC), whereas cytosolic CP for *de novo* pyrimidine synthesis is generated independently by CAD (CPS2) using glutamine as a nitrogen source; these two CP pools are functionally separate. In KRAS/LKB1-mutant NSCLC cells, however, a portion of CPS1-derived mitochondrial CP is rerouted from the urea cycle to the cytosol, where it enters the pyrimidine biosynthesis pathway, providing an alternative CP pool that sustains pyrimidine availability for DNA replication. This cross-compartmental rerouting was confirmed by ^15^N tracing, which showed CPS1-dependent transfer of ammonia-derived nitrogen into thymidine (Kim et al., 2017). Consequently, silencing CPS1 in KL cells causes pyrimidine depletion, replication fork stalling, and DNA damage, and exogenous pyrimidines fully rescue these defects. Thus, the regulatory node linking ammonia metabolism to pyrimidine synthesis in KL cells is the mitochondrially-generated CP from CPS1, whose diversion from the urea cycle to the pyrimidine *de novo* pathway becomes essential for nucleotide homeostasis under LKB1 loss and KRAS mutation. Furthermore, research has concentrated on oncogenes and tumor suppressor genes, with mutations in oncogenes modulating ammonia metabolism to varying degrees, impacting cancer cell proliferation. p53 deficiency results in elevated expression of urea cycle enzymes (CPS I, OTC, and ARG1), thereby promoting ammonia metabolism and urea production. p53 represses these genes transcriptionally, thereby affecting urea production and ammonia clearance (Nigro et al., 1989; Vogelstein et al., 2000; Olivier et al., 2010). In p53-deficient cells, the accumulation of ammonia promotes polyamine synthesis and influences cell proliferation by inhibiting ornithine decarboxylase (ODC), the rate-limiting enzyme in polyamine biosynthesis. Accumulating evidence indicates that three key oncogenic drivers (c-MYC, hypoxia-inducible factor 1α (HIF1α), and p53) orchestrate tumor cell behavior through the regulation of energy metabolism, with ammonia metabolism emerging as a central node within this network (Dai et al., 2020). Earlier work by Chiodi et al. demonstrated that tumor cells develop a dependency on glutamine (Chiodi et al., 2021). Subsequent studies under glutamine-deprived conditions revealed that c-MYC promotes tumor proliferation by upregulating GLUL, thereby enabling aspartate utilization to sustain growth. Mechanistically, the transcription factors encoded by c-MYC, HIF1α, and p53 directly regulate genes involved in ammonia metabolism, such as GLUL, to drive metabolic reprogramming in cancer cells. Some of these genes target rate-limiting enzymes in ammonia handling, exerting profound effects on tumor survival and progression.

In pancreatic cancer mouse models, GLUL expression is upregulated in both cancer cells and the tumor microenvironment and is associated with disease progression (Bott et al., 2019). Genetic depletion of GLUL delays tumor onset and prolongs survival, suggesting that GLUL-mediated nitrogen recycling may support pancreatic tumorigenesis in selected contexts. Mechanistically, GLUL converts ammonia into glutamine, thereby helping maintain glutamine-dependent biosynthesis under nutrient-limited conditions (Bott et al., 2015). Cell culture studies further show that GLUL supports proliferation during glutamine deprivation and contributes to nucleotide and amino sugar biosynthesis through ammonia reassimilation (Cox et al., 2016). However, the dependence on GLUL appears to be context-specific and should not be generalized to all tumors. Together, these findings suggest that ammonia metabolism may represent a genotype- and microenvironment-dependent metabolic adaptation shaped by oncogenic signaling rather than a uniform vulnerability across cancers. Future studies should clarify the regulatory circuits linking KRAS, LKB1, p53, c-MYC, and GLUL-dependent ammonia handling, and evaluate whether these dependencies can be exploited through biomarker-guided metabolic therapies.

## 4 Role of ammonia metabolism in tumor immune microenvironment

### 4.1 Influence of ammonia metabolism on T cells

The tumor microenvironment is frequently characterized by hypoxia, nutrient deprivation, and accumulation of metabolic by-products, which together impose metabolic stress on infiltrating T cells. Dysregulated nitrogen metabolism and ammonia accumulation may contribute to impaired T-cell immunity in selected tumor contexts. In colorectal cancer mouse models, metastatic TripleMut mice exhibit reduced T-cell numbers and impaired T-cell function compared with SingleMut mice, a phenotype associated with ammonia accumulation (Bell et al., 2023; Liao et al., 2019). Mechanistically, increased ammonia can suppress CD4^+^ and CD8^+^ T-cell proliferation, reduce activation markers such as CD25, and increase exhaustion-associated markers including PD-1 and TIM-3 (Bell et al., 2023; Bian et al., 2020; Chang and Pearce, 2016; Sinclair et al., 2019). Ammonia may also disrupt sulfur amino acid metabolism by limiting transsulfuration-dependent conversion of methionine to cysteine, thereby reducing glutathione synthesis and weakening redox homeostasis in effector T cells (Bian et al., 2020; Chang and Pearce, 2016). In addition, elevated intracellular ammonia has been linked to mitochondrial dysfunction, impaired oxidative phosphorylation, increased reactive oxygen species production, and reduced bioenergetic fitness, all of which may compromise sustained T-cell activation and proliferation. Domagala et al. further showed that ammonia impairs T-cell cytotoxicity by suppressing perforin maturation, a process associated with lysosomal alkalinization (Domagala et al., 2025). Because neutral ammonia (NH_3_) can diffuse across membranes and accumulate within acidic organelles, it may selectively increase lysosomal pH without necessarily altering the overall acidity of the tumor microenvironment (Voskoboinik et al., 2015; Lopez et al., 2012). This compartment-specific lysosomal alkalinization can interfere with perforin conformational maturation, thereby weakening T-cell-mediated tumor killing. Collectively, these findings suggest that ammonia accumulation may contribute to T-cell dysfunction and immune evasion in certain tumor settings, although its relative contribution compared with other metabolic stressors in the tumor microenvironment requires further investigation.

Nutrient competition represents an additional metabolic pressure that shapes T-cell function within the tumor microenvironment. Tumor cells and infiltrating T cells compete for glucose and amino acids required for nucleotide synthesis, biosynthesis, and effector differentiation, including glutamine, glycine, serine, arginine, and branched-chain amino acids (BCAAs) (Lim et al., 2020; Delgoffe, 2016; DePeaux and Delgoffe, 2021; Wetzel et al., 2023). Because activated CD4^+^ and CD8^+^ T cells have high nutrient demands, depletion of these substrates can impair T-cell activation, clonal expansion, and cytotoxic function. For example, arginine depletion mediated by arginase activity can restrict T-cell activation and contribute to an immunosuppressive microenvironment (Líndez et al., 2019). This effect may be particularly relevant in cancer-associated metabolic stress states, where systemic or local arginine availability is reduced and immune-cell demand exceeds endogenous production (Geiger et al., 2016; Poillet-Perez et al., 2018; Riess et al., 2018). Similarly, glutamine and BCAAs are required for T-cell metabolic fitness, and their limited availability can compromise T-cell expansion and effector differentiation (Sinclair et al., 2013). However, these nutrient-competition pathways should be distinguished from ammonia-specific mechanisms and considered as related metabolic stressors that may cooperate with ammonia accumulation to weaken antitumor T-cell immunity.

Ammonia accumulation has recently been proposed as an important metabolic stressor that may influence T-cell fate within the tumor microenvironment in a cell type dependent manner. Tumor cells with dysregulated nitrogen metabolism or impaired urea-cycle activity can release elevated levels of ammonia, which may impose a chronic metabolic burden on infiltrating lymphocytes (Bell et al., 2023). Effector T cells, which require active mitochondrial respiration, amino acid metabolism, and redox balance to sustain proliferation and cytotoxicity, appear particularly vulnerable to ammonia-associated stress. Mechanistically, excess ammonia may perturb intracellular nitrogen homeostasis and accumulate within acidic organelles, leading to lysosomal alkalinization and impaired degradative capacity. This disturbance can compromise autophagic flux and secondarily affect mitochondrial function, as reflected by reduced oxidative phosphorylation, increased reactive oxygen species, and impaired bioenergetic fitness. In parallel, ammonia may interfere with sulfur amino acid metabolism by suppressing transsulfuration flux, resulting in reduced cysteine and glutathione availability and further aggravating oxidative stress. Together, these effects have been linked to a noncanonical ammonia-induced effector T-cell death phenotype, termed “ammonia death” or “ammonoptosis” (Zhang H. et al., 2024). In the reported models, ammonoptosis appears to differ from classical apoptosis, pyroptosis, necroptosis, ferroptosis, and autophagy-dependent cell death, as it is not primarily characterized by canonical caspase activation, gasdermin D cleavage, RIPK1/RIPK3–MLKL signaling, lipid peroxidation, or typical autophagic cell death markers (Kayagak et al., 2015; Dixon and Olzmann, 2024; Shi et al., 2017; Weindel et al., 2022). Instead, this phenotype has been associated with lysosomal alkalinization, impaired autophagic flux, secondary mitochondrial dysfunction, ATP depletion, and plasma membrane disruption. However, ammonoptosis remains an emerging concept, and whether it represents a distinct and broadly conserved form of regulated T-cell death, or rather a context-specific consequence of ammonia-induced organellar stress, requires further validation across tumor types and immune-cell states.

Regulatory T cells may respond differently to ammonia-rich conditions. Compared with effector T cells, Tregs may possess greater metabolic adaptability and may engage nitrogen-handling pathways related to urea-cycle-associated enzymes and polyamine biosynthesis, potentially supporting their survival and suppressive function under metabolic stress (Gu et al., 2025). This metabolic flexibility could contribute to the relative persistence of immunosuppressive Treg populations in ammonia-rich tumor niches. Nevertheless, whether ammonia directly promotes Treg expansion or instead selects for metabolically adapted Treg subsets remains unresolved. Therefore, ammonia should be interpreted as a potential metabolic pressure that may differentially affect effector T cells and Tregs, rather than as a fully established driver of Treg dominance or immune escape.

In addition to the noncanonical ammonoptosis described above, ammonia can also promote classical apoptosis in immune cells through several distinct mechanisms, as detailed below. Ammonia may promote immune cell apoptosis aside from ammonia death through several mechanisms including which ammonia activates the TNF-α/TNFR1 signaling pathway, triggering the death receptor-mediated apoptosis. The binding of ammonia to TNF-α activates its death domain, recruits TRADD and FADD proteins, forms a complex, activates Caspase-8, and subsequently Caspase-3, leading to apoptosis (Wang et al., 2020). Ammonia induces apoptosis *via* the mitochondrial pathway. It downregulates mitochondrial membrane potential, causing pro-apoptotic factors such as Bax, Bid, and Bak to bind to the mitochondrial membrane, disrupt it, release Cytc, and activate Caspase-9, which then activates Caspase-3, inducing cell death (Lin et al., 2021). Ammonia reduces miR-27b-3p expression, enhancing apoptosis-related gene expression (e.g., TRADD, FADD, APAF-1), thus promoting both death receptor and mitochondrial pathway activation (Zhang et al., 2022; Chen et al., 2019). Ammonia exposure alters immune cell cytokine secretion, causing an imbalance in immune responses. Specifically, cytokines secreted by Th1 and Treg cells (e.g., IFN-γ, IL-2) decrease, while those from Th2 and Th17 cells (e.g., IL-1β, IL-4, IL-6) increase, suppressing immune function and promoting apoptosis (Lee and Kim, 2017). Ammonia exposure activates heat shock proteins (HSPs) like HSP 25, HSP 40, and HSP 70, which regulate immune factor expression and may play a key role in immune cell apoptosis and immune suppression (Magouz et al., 2021; Liu et al., 2021).

Recent studies suggest that ammonia may contribute to immune evasion by enhancing tumor-cell stress adaptation and reducing susceptibility to T cell–mediated cytotoxicity in selected contexts (Eng et al., 2010; Moreno-Sánchez et al., 2020). Ammonia concentrations detected in tumor-cell culture supernatants are comparable to levels previously shown to induce autophagy, supporting the view that ammonia may function not only as a toxic by-product but also as a bioactive metabolite involved in stress responses. Mechanistically, glutamine withdrawal can induce rapid apoptosis in Myc-transformed cells, whereas ammonia released from glutamine-catabolizing tumor cells may act in an autocrine or paracrine manner to stimulate autophagy and promote survival under nutrient-limited or hostile microenvironmental conditions (Wise et al., 2008; Gao et al., 2009; Yuneva et al., 2007). Although a similar role for ammonia as a diffusible metabolic signal has been described in budding yeast, whether ammonia-induced autophagy directly protects tumor cells from T cell–mediated killing in human tumors remains speculative and requires direct experimental validation (Palková et al., 1997).

T-cell activation and antitumor function are also shaped by broader metabolic programs within the tumor microenvironment. Glutaminase activity, leucine-dependent mTORC1 signaling, and SLC1A5-mediated glutamine transport contribute to T-cell differentiation and effector function, including TH1, TH17, and cytotoxic CD8^+^ T-cell responses (Johnson et al., 2018; Na et al., 2014). In parallel, immune checkpoint pathways such as PD-1 and CTLA-4 suppress effector T-cell metabolism by reducing AKT signaling, amino acid uptake, and overall metabolic activity (Bott et al., 2015; Chamoto et al., 2017; Chang et al., 2015; Zhang et al., 2017; Patsoukis et al., 2015; Chang et al., 2013). Tryptophan depletion may further impair the antitumor function of infiltrating lymphocytes. These pathways provide important context for metabolic immune regulation, but they should be distinguished from ammonia-specific mechanisms. Thus, ammonia-associated autophagy and nutrient-dependent T-cell dysfunction may cooperate to weaken antitumor immunity, although the direct contribution of ammonia to immune escape remains to be defined.

**FIGURE 4:**
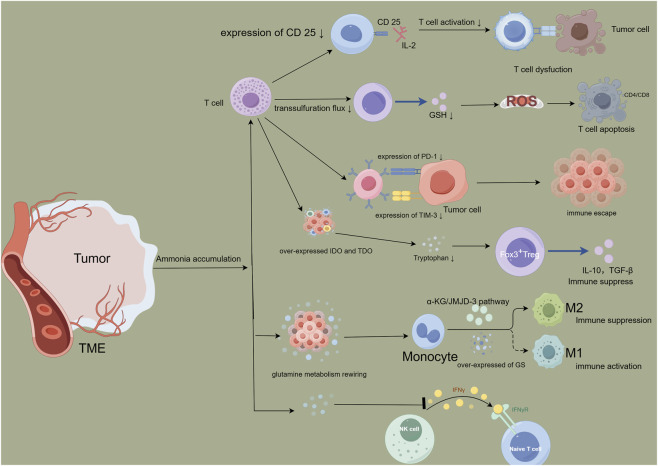
Impact of ammonia accumulation on immune cells within the tumor microenvironment and its potential mechanisms. (Ammonia dampens effector T cell activation by reducing CD25/IL-2 signaling and constraining transsulfuration-dependent glutathione production, thereby elevating ROS and promoting T cell dysfunction and apoptosis. In parallel, ammonia reinforces exhaustion programs by enhancing inhibitory checkpoint signaling (PD-1 on T cells and TIM-3 on tumor cells). Within the tumor microenvironment, sustained ammonia accumulation acts as a metabolic cue that reshapes immune cell fate by reprogramming nitrogen utilization pathways. In monocytes and macrophages, ammonia-associated glutamine metabolic rewiring induces elevated expression of glutamine synthetase, enhancing nitrogen reassimilation and redirecting intracellular metabolic flux. Glutamine synthetase-dependent metabolic adaptation increases α-ketoglutarate availability and engages α-KG–JMJD3–dependent epigenetic programs, thereby promoting polarization toward an immunosuppressive M2-like phenotype while constraining pro-inflammatory M1 differentiation. This metabolic–epigenetic coupling attenuates antigen presentation and inflammatory signaling, weakening antitumor immune activation. In parallel, ammonia-enriched conditions cooperate with tumor-driven tryptophan catabolism, mediated by IDO and TDO, to favor the expansion and functional stabilization of Foxp3^+^ regulatory T cells (Tregs). Foxp3^+^ Tregs exert potent immunosuppressive effects through sustained secretion of the anti-inflammatory cytokines IL-10 and TGF-β, which suppress effector T cell activation, proliferation, and cytotoxicity, and further dampen antigen-presenting cell function. Collectively, ammonia-driven myeloid polarization and Treg-mediated cytokine signaling establish a reinforcing immunosuppressive network that promotes immune evasion and supports tumor progression. In addition, altered nitrogen/glutamine metabolism attenuates NK cell IFN-γ production and weakens IFN-γ receptor–mediated priming of naïve T cells. Collectively, these metabolically driven immune defects establish an immunosuppressive TME that enables immune evasion and disease progression. Figure created by Figdraw).

Modulating amino acid levels within the TME represents a potential strategy for tumors to regulate immune cell function. Cancer cells, tumor-associated macrophages, and certain dendritic cells can deplete local tryptophan levels by upregulating metabolic enzymes, including indoleamine 2,3-dioxygenase (IDO1) and tryptophan 2,3-dioxygenase (TDO2) (Fallarino et al., 2006). Increased activity of tryptophan catabolic enzymes promotes the conversion of tryptophan to its metabolite kynurenine (Matés et al., 2020; Munn et al., 2002; Uyttenhove et al., 2003). The depletion of tryptophan in the local environment impairs effector T cell function, while kynurenine degradation products can suppress tumor immunity by inducing the generation of Foxp3+ regulatory T cells (Tregs) (Sharma et al., 2007). Recent studies indicate that alanine is crucial for early T cell activation and the re-stimulation of memory CD8^+^ T cells. However, due to the limited expression of ALT1/2 (or GPT1/2) and low transaminase activity, which restricts alanine biosynthesis, T cells primarily rely on extracellular alanine for protein synthesis (Ron-Harel et al., 2019). This suggests that during nutrient starvation, the consumption of extracellular alanine by cancer cells negatively impacts T cell function. The metabolic composition of the TME significantly influences both tumor cells and infiltrating T cells. Glutamine-derived nitrogen is essential for the clonal expansion and differentiation of activated T cells into effector cells (Sinclair et al., 2013; Wang et al., 2011). However, limiting glutamine consumption through SLC1A5 deficiency or restricted local glutamine availability can promote the expression of Foxp3, a key transcription factor for Treg lineage specification (Na et al., 2014; Metzler et al., 2016). Decreased β-lactam degradation releases α-KG-dependent demethylation at the Foxp3 locus, which promotes the generation of suppressive Tregs and inhibits TH1 differentiation (Klysz et al., 2015). Another study suggests that the accumulation of 2-HG (2-hydroxyglutarate) is likely due to increased transamination activity mediated by glutamate oxaloacetate transaminase(GOT1), resulting in promoter methylation at the Foxp3 locus and reduced TcB induction (Xu et al., 2017). Furthermore, glutamine is a precursor for glucosamine synthesis, which is essential for protein glycosylation and has been shown to be critical for activated T cell function (Swamy et al., 2016; Araujo et al., 2017). Therefore, increased glutamine consumption by cancer cells can modulate anti-tumor immunity by depleting the local glutamine pool required for effector T cell responses, while promoting the development of suppressive Treg populations. Interestingly, a recent report showed that while cancer cells are sensitive to glutamine antagonism, effector T cells can redirect their metabolism towards a more oxidative, long-lived activation phenotype (Leone et al., 2019). As detailed in Figure 4, Table 2.

**TABLE 2:** The mechanisms by which ammonia accumulation in the tumor microenvironment impairs immune cell function (This table summarizes the extent of immune cell dysfunction under different amino acid concentrations in various tumor microenvironments along with the potential mechanisms involved. PP: Peyer’s patches).

Immune cell	Study material	Study result	Ammonia exposure	Possible mechanism	References
CD8^+^ Teff	CD45.1^+^CD8^+^ OT-I T cell (in C57BL/6J)	Percentage of T cell declines	20–30 µM ammonia in cytoplasm	Ammonia transport induces lysosomal damage, followed by mitochondrial ammonia retention and impaired clearance of damaged mitochondria, leading to cell death.	Domagala et al. (2025)
	OT-I Teff (*in vivo*)	Area of propidium iodide (PI) staining	10–20 µM NH_4_CL in medium		
Human T cell	T cell in HCT116 cell line	Expression of PD-1 incline	5/10 mmol ammonia in medium	Ammonia preferentially inhibits T cell growth mainly by inducing T cell exhaustion and upregulating surface immunosuppressive markers.	Bell et al. (2023)
CD3^+^ T cell	SingleMut Mice (*in vivo*)	Expression of PD-1/CTLA-4 incline Expression of IFNγdecline	ammonium acetate diet		
CD3^+^ T cell	Human T cell in HCT116,RKO,SW480,DLD1	Expression of PD-1, CTLA-4 incline Expression of CD25 and precentage of T cell decline	Exposure in 5-10um ammonia in medium in 72h		
CD4^+^ T cell					
CD8^+^ T cell					
NK cell	Human PBMC	Area of propidium iodide (PI) staining and ADCC on different tumor cells	1–5 mmol NH4CL in medium	Ammonia induces NK and T cell dysfunction by reducing the secretion of mature perforin in lysosomes.	Domagala et al. (2025)
CD19 CAR T cell					
Macrophage	yellow catfish	cell viability of macrophage	0.12/0.23/0.46 mg/L ammonia in medium	Ammonia stress triggered ferroptosis in the macrophages	Gan et al. (2022)
B cell	PP in F/A-2+/+ Mice	Percentage of B cell/Expression of CD19, I-A declines	80 µM Arg in blood	Arginase-related gene mutations lower arginine levels in the microenvironment, inhibiting B cell function.	De Jonge et al. (2002)

### 4.2 Influence of ammonia metabolism on TME stromal cells

In in vitro cell line experiments, high-glutamine culture conditions show that fibroblasts enhance mitochondrial biosynthesis in cancer cells (e.g., MCF7 cells) by providing glutamine, thereby promoting cancer cell survival (Ko et al., 2011). Fibroblasts help cancer cells resist apoptosis and maintain metabolic stability by regulating Cav-1 expression, increasing glutamine transporters (SCF family), and activating autophagy. Moreover, glutamine helps cancer cells reduce apoptotic risk by upregulating TIGAR (TP53-induced glycolysis and apoptosis regulator, TIGAR) protein and may also contribute to drug resistance, indicating that the synergy between glutamine and fibroblasts plays a critical role in cancer cell growth and resistance. Cancer-associated fibroblasts (CAFs) modulate glutamine metabolism in the tumor microenvironment through multiple mechanisms, promoting tumor cell growth, proliferation, and metastasis. CAFs secrete glutamine, providing a nitrogen source essential for tumor cells, thereby supporting rapid proliferation. They also enhance the uptake and metabolism of glutamine in tumor cells, satisfying their elevated metabolic needs.

CAFs also influence tumor cell energy and biosynthetic pathways by regulating the activity of enzymes involved in glutamine metabolism (Li et al., 2021; Yang et al., 2016). CAFs secrete cytokines such as TGF-β, which alter the acidic state of the tumor microenvironment (Reid et al., 2013; Abulaiti et al., 2013; Vanhove et al., 2019). This may exacerbate acidification by promoting the accumulation of lactate and ammonia, further supporting tumor cell adaptability and invasiveness. Tumor cells often undergo metabolic reprogramming, enabling them to adapt to hypoxia, acidity, and nutrient scarcity within the microenvironment. CAFs play a role in tumor cell migration and invasion by modulating metastasis-related signaling pathway, such as the mTOR and AMPK pathways, thereby enhancing the metastatic properties of tumors (Nicklin et al., 2009; Yang et al., 2009). CAFs secrete proteins that regulate ammonia metabolism. Through the secretion of cytokines like transforming growth factor β (TGF-β), matrix metalloproteinases (MMPs), and other metabolic products, CAFs alter the metabolic pathways of tumor cells (Zhang et al., 2022). These factors not only influence tumor cell growth but also modulate the activity of glutamine metabolism networks. Through these mechanisms, CAFs enhance tumor cell demand for and uptake of glutamine (Abulaiti et al., 2013; Kurt-Celep et al., 2022). Additionally, CAFs may play a role in remodeling the tumor microenvironment by secreting glutamine and other molecules, which enhance the survival of tumor cells and promote their dissemination and invasion. For other stromal cells, like endothelial cells, glutamine metabolism is vital. Disruption of this process through glutaminase, glutamine synthetase, or glutamine starvation can impair vascularization *in vivo*.

### 4.3 Influence of ammonia metabolism on macrophages in TME

In human macrophages, nutrient deficiency induces the expression of GLUL, which supports the pro-angiogenic, immunosuppressive, and pro-metastatic capabilities of macrophages (Sousa et al., 2016). Chemical inhibition of GLUL enhances several markers of pro-inflammatory macrophages. These findings align with the role of glutamine in the differentiation of pro-inflammatory macrophages in mouse models (Liu et al., 2017). Specifically, inhibition of GLUL or glutamine deprivation results in increased succinate levels and stabilization of HIF 1α, a key transcription factor in pro-inflammatory macrophages (Palmieri et al., 2017). Macrophages not only play a central role in immune responses but also regulate tumor malignancy through their polarization state. Macrophages are typically categorized into M1 and M2 types. M1 macrophages have pro-inflammatory and anti-tumor effects, induced by Th1 cytokines such as interferon-λ (IFN-λ) and interleukin-1β (IL-1β). In contrast, M2 macrophages exhibit immunosuppressive, pro-tumor, and anti-inflammatory properties, typically activated by IL-4 and IL-13. In tumor settings, the expression of GLUL is often regulated. Targeting GLUL shifts macrophages toward an M1-like phenotype, leading to enhanced CD8^+^ T cell accumulation and reduced tumor metastasis (Cobbold et al., 2009; Serafini et al., 2008). In the polarization of M2-type tumor-associated macrophages (TAMs), glutamine promotes M2 activation through its metabolite α-KG and influences metabolic reprogramming *via* the JMJD-3 dependent pathway (Liu et al., 2017). Regulation of glutamine metabolism plays a crucial role in the transition of TAMs to an immunosuppressive phenotype, fostering tumor immune evasion and progression. Additionally, ornithine, through ODC, is converted to polyamines, which are critical for M2 polarization. Polyamines stimulate the expression of M2-related genes, maintaining their pro-tumor phenotype. Polyamine production also suppresses M1-type TAM activation, further promoting tumor growth and immune evasion (Van Den Bossche et al., 2012; Hardbower et al., 2017).

An expanding body of evidence indicates that ammonia is not merely a passive metabolic waste product in cancer, but a bioactive metabolite that can reshape nitrogen metabolic circuitry and organellar homeostasis, thereby profoundly modulating macrophage immune function within the tumor microenvironment (Xu et al., 2025). In macrophages, ammonia-driven metabolic rewiring appears to converge on arginine-centered metabolic branchpoints. Macrophage effector states are critically governed by the balance between the iNOS–NO axis, which supports pro-inflammatory and antitumor immunity, and the ARG1–ornithine–urea/polyamine axis, which is closely linked to immunosuppression and tissue-repair phenotypes. Increased ammonia burden and redistribution of nitrogen flux favor an ARG1-dominant program, enhancing ornithine and polyamine biosynthesis and promoting polarization of tumor-associated macrophages toward an immunosuppressive state (Murray, 2017). In parallel, ammonia-associated metabolic stress can couple to mitochondrial metabolism and redox control, attenuating iNOS activity and nitric oxide production and dampening antigen presentation and pro-inflammatory signaling. Beyond these direct metabolic effects, ammonia-driven macrophage reprogramming may further suppress T cell and NK cell function indirectly through arginine depletion, release of immunosuppressive mediators, and remodeling of the local metabolic milieu. Collectively, these findings position ammonia as a metabolic signaling molecule that integrates nitrogen handling, arginine pathway regulation, and immunoregulatory networks to reinforce an immunosuppressive tumor microenvironment and cooperate with effector lymphocyte dysfunction to facilitate tumor immune escape.

### 4.4 Influence of ammonia metabolism on NK cells in TME

In the context of tumor immunity, ammonia accumulation plays a critical role in inhibiting the cytotoxic function of natural killer (NK) cells, especially in the hypoxic tumor microenvironment (Presnell et al., 2020). This metabolic product also impedes the migration of NK cells to the tumor site, further weakening their anti-tumor activity. Ammonia inhibits the secretion of key immune factors, such as interferon-γ (IFN-γ), which are crucial for an effective anti-tumor immune response. NK cell-mediated target cell killing involves several steps, including activation, conjugation with the target cell, degranulation, cytokine production, and detachment from the target cell. The combined effects of ammonia accumulation and hypoxia not only enhance immune suppression but also promote tumor immune escape, fostering the tumor’s resistance to immune surveillance and therapeutic interventions. Targeting ammonia metabolism may therefore offer a promising approach to enhance NK cell-based cancer immunotherapy.

**FIGURE 5:**
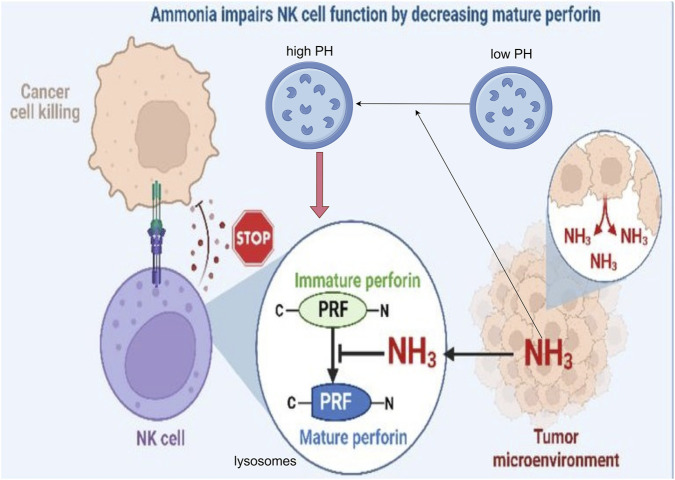
The maturation process of perforin is influenced by intracellular ammonia. (NH_3_ diffusion perturbs NK cell pH homeostasis, elevating lysosomal pH. Because perforin (PRF) requires an acidic lysosomal environment for proteolytic processing into its mature, pore-forming form, ammonia-driven alkalinization inhibits PRF maturation, leading to accumulation of immature PRF and depletion of mature PRF. Consequently, NK cell degranulation potency and target-cell lysis are reduced, weakening innate immune surveillance and facilitating tumor immune evasion. Reprinted from Cancer Research, Vol 85, Domagala, Joanna et.al, ammonia Suppresses the Antitumor Activity of Natural Killer Cells and T Cells by Decreasing Mature Perforin, Pages No 2464., Copyright (2025), with permission from American Association for Cancer Research).

Recent literature suggests that ammonia also modulates NK cell function *via* perforin, a crucial protein involved in the cytotoxic killing of target cells by NK cells (Domagala et al., 2025). *In vitro* experiments showed that under high ammonia concentrations, the levels of NK cell surface activation receptors (NKG2D, NKp30, NKp44, NKp46) were unaffected, and the degranulation process remained unchanged (Domagala et al., 2025). Ammonia inhibits perforin maturation, leading to reduced levels of mature perforin in NK cells and consequently diminishing NK cell cytotoxicity. Specifically, ammonia impairs the conversion of perforin from its precursor to the mature form, disrupting its normal function. Perforin maturation and function are pH-dependent within the lysosome. Ammonia raises the lysosomal pH, interfering with perforin maturation. Perforin typically transitions from its precursor to its active form in a low-pH environment. Ammonia accumulation weakens the acidic environment of the lysosome, preventing effective perforin maturation or causing its degradation, thus diminishing its cytotoxic activity. Ammonia not only reduces the level of mature perforin in NK cells but also alters the distribution of other lysosomal markers (e.g., LAMP-1). *In vitro* experiments confirmed that ammonia’s inhibition of perforin is highly concentration-dependent, with concentrations of 4–5 mM completely inhibiting perforin activity, and the inhibitory effect increasing at lower concentrations, as detailed in Figure 5. Beyond effects on perforin, we noted that the availability of glutamine, the upstream substrate of ammonia metabolism which constitutes a fundamental metabolic prerequisite for NK-cell cytokine responsiveness (Terrén et al., 2019). In IL-2/IL-12–activated NK cells, glutamine is required to sustain c-Myc protein expression, yet this requirement does not primarily reflect glutamine’s role as a bioenergetic fuel. Rather, c-Myc in activated NK cells undergoes continuous GSK3-dependent proteasomal degradation, necessitating high rates of protein synthesis to maintain steady-state abundance (Momcilovic et al., 2018). By supporting amino acid sufficiency and translational capacity, glutamine indirectly preserves c-Myc protein levels. Glutamine deprivation, in turn, downregulates c-Myc, thereby blunting NK-cell metabolic reprogramming and compromising antitumor effector functions (Loftus et al., 2018).

Taken together, ammonia accumulation may exert cell type–dependent effects on immune and stromal components within the tumor microenvironment. Cytotoxic lymphocytes, including CD8^+^ T cells and NK cells, may share a vulnerability to ammonia-associated lysosomal alkalinization, which can impair perforin maturation and weaken cytotoxic activity. In effector T cells, ammonia may further disrupt transsulfuration-dependent glutathione synthesis, mitochondrial function, and redox balance, and an ammonia-induced noncanonical cell-death phenotype, termed “ammonoptosis”, has recently been proposed. However, whether this process represents a distinct and broadly conserved form of regulated T-cell death, and whether a similar mechanism occurs in NK cells, remains to be clarified. In contrast, regulatory T cells may possess greater metabolic adaptability through urea-cycle-related enzymes and polyamine metabolism, potentially supporting their persistence under ammonia-rich conditions. Ammonia-related nitrogen flux may also influence macrophage polarization through the ARG1–ornithine–polyamine axis, while stromal cells such as cancer-associated fibroblasts and endothelial cells may indirectly modulate ammonia-related metabolic stress through nutrient exchange and vascular remodeling. Thus, ammonia should be interpreted as a context-dependent metabolic pressure that may impair cytotoxic immunity and favor immunosuppressive states in selected tumor settings, rather than as a universal driver of immune escape.

The spatial distribution of ammonia within tumors represents another important but incompletely understood layer of metabolic heterogeneity. Integrated spatial metabolomic and transcriptomic analyses in human hepatocellular carcinoma suggest that ammonia-rich microniches may arise in regions with enhanced glutaminolysis and impaired urea-cycle activity, and these regions have been associated with enrichment of regulatory T cells and loss of effector T cells (Gu et al., 2025). Local pH may further influence ammonia toxicity, indicating that hypoxia, acidosis, and ammonia accumulation may interact to shape immune-cell stress within specific tumor regions (Dravecka et al., 2025). Ammonia heterogeneity may also differ between primary tumors and metastatic lesions. Earlier PET imaging studies reported increased ammonia uptake in several metastatic tumors, including breast cancer metastases, soft tissue sarcomas, lung tumors, melanomas, lymphomas, and prostate cancer metastases (Schelstraete et al., 1982). In liver tumors, heterogeneous repression of urea-cycle enzymes may alter ammonia detoxification and promote nitrogen redistribution toward nucleotide and amino acid synthesis (Han et al., 2026; Zou et al., 2025). In the central nervous system, ammonia can cross the blood–brain barrier and is normally detoxified by astrocytic glutamine synthetase, but whether brain metastatic tumor cells exploit or tolerate this ammonia-rich environment remains unclear (Hadjihambi et al., 2014). These findings highlight the need for spatially resolved studies to determine how ammonia distribution influences immune suppression, metastatic adaptation, and therapeutic response across tumor types.

From a clinical-translational perspective, elevated ammonia is not merely a metabolic byproduct but actively drives immune evasion and therapeutic resistance. In colorectal cancer, patients exhibit increased serum ammonia, and an ammonia-related gene signature correlates with altered T cell responses, adverse outcomes, and poor response to immune checkpoint blockade. Mechanistically, high ammonia induces T cell metabolic reprogramming, increases exhaustion markers, and decreases proliferation; conversely, pharmacological enhancement of ammonia clearance reactivates T cells, reduces tumor growth, extends survival, and improves anti-PD-L1 efficacy. Collectively, these observations establish that the functional impact of ammonia on tumor progression and immune modulation cannot be generalized without considering spatial and anatomical context. Critical knowledge gaps persist: systematic quantitative comparisons of ammonia levels between matched primary tumors and metastatic deposits are virtually absent; the expression landscape of the recently identified ammonia importer SLC4A11 across cancer cell lines and metastatic sites has not been characterized; and whether the ammonia-rich microniches identified in hepatocellular carcinoma represent a phenomenon generalizable to other cancer types remains to be determined. Addressing these gaps will require integrated spatially resolved metabolomics, high-resolution *in vivo* imaging of ammonia fluxes, and functional perturbation studies that distinguish direct effects on cancer cells from indirect effects *via* immune modulation within specific TME subregions, ultimately informing whether and how regional ammonia targeting can be leveraged as a spatially tailored precision immunotherapy.

## 5 Current inhibitors targeting ammonia metabolism and future research directions

Ammonia metabolism has potential translational relevance, but its clinical application should be interpreted cautiously because most current strategies target the broader glutamine–ammonia metabolic axis rather than ammonia alone. Dysregulated ammonia handling may provide biomarkers for tumor metabolic adaptation, whereas altered nitrogen flux may create therapeutic vulnerabilities in selected genetic and microenvironmental contexts. For example, TP53-deficient castration-resistant prostate cancer cells activate the ATF4–ASNS axis and depend on glutamine-derived glutamate for *de novo* asparagine synthesis, suggesting that GLS inhibition may enhance sensitivity to asparagine depletion strategies (Yoo et al., 2024; Lemberg et al., 2022). Glutamine metabolism has also been implicated in chemotherapy resistance by supporting mitochondrial respiration, glutathione synthesis, reactive oxygen species buffering, and drug efflux programs in paclitaxel-resistant prostate cancer models (Lee et al., 2021; Gross et al., 2014; Erb et al., 2024; Vasiliou et al., 2008; Zhu et al., 2013). These findings support the rationale for combining glutamine–ammonia metabolic inhibition with chemotherapy or amino acid depletion therapies, although the efficacy of such approaches is likely to depend on tumor genotype and metabolic state.

Combination with immunotherapy represents another important but still uncertain direction. Broad glutamine antagonists, including DON-derived prodrugs such as JHU-083 and DRP-104, can suppress glutamine-derived carbon and nitrogen flux and have shown immune-modulatory effects in preclinical models (Praharaj et al., 2024; Rais et al., 2022). However, early clinical experience indicates that clinical benefit is variable. In a phase I/II study, telaglenastat (CB-839) combined with nivolumab was well tolerated but showed modest overall activity across melanoma, clear-cell renal cell carcinoma, and non-small-cell lung cancer cohorts, with responses appearing more favorable in selected ICI-naïve clear-cell renal cell carcinoma patients (Gouda et al., 2025). Similarly, CB-839 combined with panitumumab in KRAS-wild-type metastatic colorectal cancer showed limited but informative clinical activity, and correlative analyses suggested that B-cell activation signatures and PET-based glutamine uptake may help identify responsive subgroups (Ciombor et al., 2025). These studies highlight the need for biomarker-guided patient selection rather than broad application of glutamine or ammonia-related metabolic inhibitors.

Resistance and adaptive metabolic rewiring further complicate clinical translation. Preclinical models of CB-839 resistance show a shift toward fatty acid oxidation, with increased CPT1 activity and CPT2 expression, suggesting that fatty acid oxidation may provide a bypass pathway when glutamine metabolism is inhibited (Reis et al., 2019). Computational approaches have also been used to derive ammonia metabolism-related gene signatures, such as a four-gene risk model in clear-cell renal cell carcinoma that predicted prognosis and immunotherapy response across external cohorts (Gong et al., 2026). In addition, ongoing genotype-enriched trials are testing combinations based on defined molecular contexts, such as KEAP1/NFE2L2/KRAS co-mutant non-small-cell lung cancer or DNAJB1–PRKACA fusion-positive fibrolamellar carcinoma (Galan-Cobo et al., 2019; A Phase 2 Randomized, 2020; A Phase 1b, 2023). Together, these observations suggest that future clinical development should prioritize resistance biomarkers, metabolic imaging, genotype-defined enrollment, and rational combination strategies.

Ammonia-related metabolic targeting may also enhance radiotherapy response by weakening glutamine-dependent redox buffering and cancer stem cell maintenance. In prostate cancer models, elevated glutamine metabolism supports glutathione production and protects tumor cells from radiation-induced oxidative stress and DNA damage (Mukha et al., 2021). GLS inhibition can reduce glutathione synthesis, alter cellular redox balance, and restore radiosensitivity. Because glutamine metabolism also supports cancer stem cell programs through pathways such as mTOR and Notch signaling, pharmacological or genetic GLS inhibition can reduce stemness markers, deplete ALDH-positive cancer stem-like cells, and enhance radiosensitivity in experimental models (Cojoc et al., 2015; Gorodetska et al., 2024; Püschel et al., 2021). These findings suggest that targeting the glutamine–ammonia axis may help weaken therapy-resistant cell populations, although clinical validation remains limited.

Several limitations should be emphasized. Early glutamine antagonists and GLS inhibitors, including DON and BPTES, have been limited by toxicity, potency, solubility, or selectivity, whereas CB-839 has improved pharmacological properties but has not produced uniformly strong clinical responses (Veterans Administration Cancer Chemotherapy Study Group et al., 1959). Proposed biomarkers of GLS inhibitor sensitivity, including intracellular glutamate/glutamine ratios, GAC expression, and GLS enzymatic activity, require further validation. Moreover, intrinsic and acquired resistance can emerge through metabolic bypass pathways, including pyruvate carboxylation, fatty acid oxidation, and mTORC1-dependent adaptive rewiring (Méndez-Lucas et al., 2020; Biancur et al., 2017; Tanaka et al., 2015). Therefore, the future success of ammonia-related metabolic therapies will likely depend on biomarker-guided patient selection and combinations designed to prevent metabolic compensation More informations about ammonia inhibitors can be found in Table 3.

**TABLE 3:** Clinical trials of current ammonia inhibitors.

Therapeutic target	Drug	Cancer	Treatment	Trial Number	Stage	Status	Participants	Results	References
GLS	CB-839	Colorectal Cancer	CB-839/Capecitabine	NCT02861300	Phase I	Completed	50	Adverse events were predominantly gastrointestinal	Phase I (2016)
​	CB-839	AMS	CB-839/Azacitidine	NCT03047993	Phase I	Completed	28	Adverse events occurred in 5% of participants and were mainly infection related.	Phase Ib (2017)
​	CB-839	TNBC	CB-839/Paclitaxel	NCT03057600	Phase I	Completed	52	Adverse events occurred in 21% of participants and were predominantly gastrointestinal.	A Multicenter Phase 2 Study (2017)
​	CB-839	RCC	CB-839/Everolimus	NCT03163667	Phase I	Completed	69	In the experimental group, adverse events occurred in 46% of participants, primarily anemia and neurological symptoms.	A Randomized (2017)
Arginase	INCB001158	Solid Tumors	INCB001158/Pembrolizumab	NCT02903914	Phase II	Completed	260	In the experimental group, 10% of patients receiving INCB001158 (50 mg) plus pembrolizumab achieved partial response.	Safety and Pharmacokinetics (2016)
​	INCB001158	Solid Tumors	INCB001158/Chemotherapy	NCT03314935	Phase I/II	Completed	149	The experimental group receiving INCB001158 (100 mg) plus paclitaxel achieved the highest complete and partial response rates.	A Phase 1 (2017)
​	BCT-100	Solid Tumors	PEG- BCT-100	NCT03455140	Phase I/II	Completed	49	Not disclosed	A Phase I (2018)
​	BCT-100	HCC	PEG- BCT-100	NCT00988195	Phase I	Completed	15	Not disclosed	Recombinant Human Arginase I (2009)
​	BCT-100	Solid Tumors	PEG- BCT-100	NCT02285101	Phase I	Completed	23	Not disclosed	Recombinant Human Arginase 1 (2014)
ASNS	DRP-104	Solid Tumors	DRP-104	NCT04471415	Phase I/II	Terminated	61	Not disclosed	Phase 1 and Phase 2a (2020)
​	DRP-104	NSCLC	DRP-104	NCT07249372	Phase II	Recruiting	37	The overall response rate is planned to be assessed within 2 years.	A Phase 2 Study of (2025)

TNBC, Triple Negative Breast Cancer; RCC, Renal Cell Carcinoma; AMS, Advanced Myelodysplastic Syndrome.

Future studies should focus on defining when ammonia metabolism is functionally important rather than merely associated with tumor progression. Spatial metabolomics and spatial transcriptomics can help identify ammonia-rich microniches and determine how ammonia-related enzymes and transporters, such as GDH2, GLUL, SLC4A11, and urea-cycle components, are distributed among malignant, immune, and stromal cells (Planque et al., 2023; Cilento et al., 2024; Zhao Y. et al., 2024). Patient-derived organoids, immune co-culture systems, *in vivo* CRISPR screens, and microphysiological models may further support functional validation of ammonia sensitivity, resistance, and therapeutic response. These platforms may help connect spatial ammonia heterogeneity with drug response and immune regulation, but their clinical utility still requires prospective validation.

Several emerging directions, including ammonia scavenging, ferroptosis sensitization, microbiota modulation, and noncanonical cell-death pathways, remain hypothesis-generating. SLC4A11-mediated ammonia transport has been linked to cancer stem cell features, suggesting that ammonia-lowering strategies may have therapeutic potential in selected high-SLC4A11 tumors (Elaimy et al., 2024). In addition, ammonia-associated redox stress may interact with ferroptosis sensitivity, whereas microbiota-derived nitrogen recycling may influence systemic ammonia burden and treatment response in certain cancers (Tong et al., 2024; Peng et al., 2025; Li et al., 2024; Nie et al., 2024). However, strategies such as ammonia-releasing prodrugs, pyroptosis induction, probiotics, or fecal microbiota transplantation should be presented cautiously until supported by direct mechanistic and clinical evidence. Overall, ammonia-related metabolism represents an emerging therapeutic concept, but its translation into precision oncology will require validated biomarkers, spatially resolved metabolic profiling, and carefully designed clinical trials in biomarker-enriched populations.

## 6 Conclusion

This review provides a comprehensive summary of the mechanisms by which ammonia metabolic reprogramming promotes tumor proliferation and metastasis within the tumor microenvironment over the past decade. Specifically, it highlights how mutations in oncogenes such as c-MYC, KRAS, and p53 influence the expression and metabolic activity of key enzymes involved in amino acid metabolism. These molecular changes shed light on early alterations that may occur during tumor progression, offering potential therapeutic targets for future research. Additionally, the review explores how various components of the tumor microenvironment are affected by ammonia metabolic reprogramming. It examines the impact of elevated extracellular and intracellular ammonia concentrations on immune cells, leading to functional impairment and suppressed proliferation. This phenomenon is likely linked to the tumor immune evasion mechanisms, with high PD-1 expression on T cells in ammonia-rich environments potentially contributing to immune suppression in the tumor microenvironment. This relationship warrants further investigation. The review also discusses how the high intracellular ammonia levels in NK cells reduce the release of immature perforin, thereby diminishing their cytotoxic function. Targeting ammonia metabolism with specific inhibitors, in combination with immunotherapy, holds significant promise for advancing cancer treatment. In conclusion, understanding the role of ammonia metabolic reprogramming in driving tumor progression within the tumor microenvironment could open new avenues for cancer therapies.
